# Socio-economic status and the risk of breast cancer among Nigerian women: a case control study

**DOI:** 10.11604/pamj.2022.41.175.32914

**Published:** 2022-03-04

**Authors:** Samuel Onyinyechukwu Azubuike, Louise Hayes, Linda Sharp, Adewumi Alabi, Rasaaq Oyesegun, Richard McNally

**Affiliations:** 1 Department of Public Health, Faculty of Health Sciences, National Open University of Nigeria, Abuja, Nigeria,; 2 Population Health Sciences Institute, Newcastle University, Newcastle upon Tyne, United Kingdom,; 3 Department of Radiation Biology, Radiotherapy and Radiodiagnosis, College of Medicine, Lagos University Teaching Hospital, Lagos, Nigeria,; 4 Department of Radiotherapy and Oncology, National Hospital, Abuja, Nigeria

**Keywords:** Breast cancer, socio-economic status, risk factor, women, sub-Saharan Africa, Nigeria

## Abstract

**Introduction:**

an increased risk of breast cancer associated with high socio-economic status has been reported in high income countries. A few available African studies have reported inconsistent findings using different single socio-economic measures. Our aim was to investigate the association between socio-economic status and the risk of breast cancer among Nigerian women based on a range of socio-economic status measures.

**Methods:**

we conducted a hospital-based case-control study involving participants from five hospitals in Lagos and Abuja. Women were interviewed in-person between October 2016 and May 2017 using a semi-structured questionnaire. Socio-economic status was assessed based on education, occupation, income, wealth, and socio-economic index. Multivariable logistic regression was applied in data analysis using Statistical Package for Social Sciences (SPSS) version 23. Level of significance was based on 95% confidence interval or p-values less than 0.05.

**Results:**

we recruited 379 histologically confirmed breast cancer cases and 403 controls. Following full adjustments, breast cancer risk reduced as socio-economic index increased (p for trend=0.028). Although women in the highest categories of educational attainment [Odds ratio (OR)=0.21, 95% confidence interval (CI): 0.09, 0.53], and personal income (OR=0.37, 95% CI: 0.19, 0.72) had a reduced risk of breast cancer compared to women in the lowest categories respectively after adjustments for relevant covariates, income alone exhibited a significant risk reduction following mutual adjustment for other socio-economic status measures (p for trend=0.014).

**Conclusion:**

the observed associations between high socio-economic status and lower breast cancer risk in Nigeria contrast with predominant findings in high-income countries. It suggests the need for socio-economic intervention and other preventive programmes such as improved access to screening and diagnostic services targeted at women of low socio-economic status in Nigeria.

## Introduction

Evidence of rising incidence of breast cancer has been reported in Africa [1]. In Nigeria (the most populous country in Africa), an estimated 26,310 new cases occurred in 2018 [2]. This was projected to increase by approximately 4000 cases per annum over the next 10 years [3]. It is unclear whether this trend might be attributed to increased exposure to putative breast cancer risk factors, increasing life expectancy, population ageing or improved detection of incident cases.

One factor that has been rather consistently associated with risk of breast cancer in other parts of the world is socio-economic status (SES) [4,5]. High SES, measured in various ways including area-based measures (e.g. deprivation), and individual-based measures (e.g. women´s own level of educational attainment, income and their husbands´ occupation) has been associated with higher breast cancer risk especially in high income countries (HIC) [4,6]. In Nigeria, women´s participation in society has been changing and with it the distribution of socio-economic status. The National Demographic Health Survey (NDHS) data since 1990 [7-10] have indicated changes in the level of educational attainment, occupational status and income generation among Nigerian women. For example, the number of women completing secondary education increased from 18.9% in 1990 to 44.8% in 2013 while the number of women employed in any occupations rose from 49.7% in 1990 to 59.8% despite a high poverty rate [7,11-13]. The distribution of these variables varied between urban and rural areas, as well as across cities and geopolitical zones, with the population of women completing secondary education, and those in employment being higher in the urban areas, southern regions and key cities such as Abuja and Lagos.

To date, the association between socio-economic status and the risk of breast cancer in Africa has only been reported in studies based on single measures of SES such as education and property index [14]. The findings of these studies, however, were not only inconsistent but were not adjusted for the effects of relevant explanatory variables such as age at first birth, parity, breastfeeding and physical inactivity. The aim of this study was to investigate the association between SES and breast cancer risk based on a range of SES measures.

## Methods

**Study design and setting:** a hospital-based case-control study was conducted in four public tertiary hospitals (University of Lagos Teaching Hospital (LUTH), Lagos State University Teaching Hospital (LASUTH), University of Abuja Teaching Hospital, Gwagwalada-UATH, National Hospital Abuja (NHA) and one secondary health facility (General Hospital, Lagos Island-GHLI). Lagos (Southern Nigeria) and Abuja (Northern Nigeria) are the two most important cities in Nigeria being the former and current federal capital city respectively. With a population of more than 12.5 million in 2016, Lagos is the largest city in sub-Saharan Africa, and projected to be the largest city in the world by 2100 [15]. Abuja has witnessed a huge population growth since 1991 and is currently listed among the world´s fastest-growing cities with more than 3.5 million people in 2016 [16]. The two cities were selected to enhance the external validity of the results owing to their rich population diversity in terms of ethnicity and socio-economic status [17]. Hospital attendance in Nigeria is not strictly guided by referral policies and catchment location because patients bear the financial cost of their treatment in both public and private hospitals [18]. Available data suggest that most cases of breast cancer in Nigeria (>86%) are diagnosed in tertiary hospitals. Moreover, some women who are initially diagnosed in private hospitals are referred to public tertiary hospitals owing to availability of better equipment and specialised staff. These considerations, resource constraints and the fact that similar design have been applied in previous indigenous studies within the study location informed the choice of the study design.

**Study population:** the study population comprised incident cases of female breast cancer attending public hospitals (especially tertiary care) between July 2015 and March 2017. Available records suggest that about 700 cases of breast cancer were seen across public tertiary health care facilities in Lagos and Abuja the year prior to the beginning of the study.

**Sample size:** the suitability of the sample size was confirmed based on the data available from a previous Nigerian study [19], using the formula [20] below.


$$\frac{r+(P*)(1-p*){\left(Z_{\beta }+Z_{\alpha }/2\right)}^{2}}{r{\left(p_{1}-p_{2}\right)}^{2}}$$

r = ratio of controls to cases, p1 represents proportion of cases, while p2 represents the proportion of controls exposed to putative risk or protective factors, p* represents the average proportion of cases and controls exposed to the putative risk or protective factor, Z_β_ represents the standard normal deviate for power of 80%, while Z_α/2_ represents the standard normal deviate for 95% confidence interval. Allowance of 20% non-response rate was made.

**Sampling technique, selection criteria, recruitment:** all eligible cases who were receiving treatment at the participating hospitals within the period of the study were consecutively sampled in proportion to the population of cases available in each study site. This was done in the order in which the patients arrived at the clinics each day as shown in the attendance register. This was the process acceptable to the oncology departmental heads who did not permit any contact with patients outside of clinic hours for recruitment purpose citing ethical concerns. No access was possible to the attendance registers in the ophthalmology clinics (source of controls), so controls were consecutively sampled based on the order of arrival/sitting in the waiting area. We adopted this approach because we have no evidence that the order of attendance and sitting during clinic hours was associated with socio-economic status. Moreover, a similar method has been applied in a previous study.

Cases were women, aged 20-80 years, diagnosed with histologically confirmed invasive breast cancer who attended oncology clinics in the oncology departments of the participating hospitals between October 2016 and May 2017. All cases whose date of diagnosis had exceeded 18 months at the time of interview were excluded to reduce information bias due to forgetfulness. The original intention was to include cases who were diagnosed not more than 12 months before the interview, but because of difficulties in confirming dates of diagnosis pre-interview, some participants (16.4%) were diagnosed 13-18 months before the interview. Controls were women who attended outpatient ophthalmology clinics in the ophthalmology departments of the same hospitals during the same period. The ophthalmology departments offered comprehensive eye services involving preventive, curative, and rehabilitative services. Hence, they attracted people of all SES. Controls were either female ophthalmology patients or female relatives (visitors) [14] aged 20-80 years, who had no personal history of breast cancer or breast disease. However, where a patient´s close relative was selected as a control, the patient was no longer eligible, and vice versa. This was because the patients were assumed to have similar exposure patterns to their female relatives. A small number of controls (4%) were recruited from the General out-patient department clinics (GOPD) to complete the required sample of controls. Frequency matching (based on age) was used to match potential controls to cases. This was done by grouping cases within age intervals of 5 years. At least an equal number of eligible controls whose ages fell within specific case age intervals were recruited. All participants were considered, by collaborating physicians, to be physically and psychologically able to participate. These collaborating physicians did not participate in the interviewing of the participants to reduce interviewer bias.

The study aims and what participation would involve were explained to potential participants (both cases and controls) during clinic hours. Afterwards, interviewers (comprising doctors, nurses, and graduates of related fields) approached the potential participants in the waiting area to confirm their eligibility and willingness to participate in the study.

**Data collection procedure:** all eligible participants who provided written or oral consent were interviewed in person using a semi-structured questionnaire. The instrument was developed specifically for the study based on information from previously validated questionnaires, taking local context into consideration [5,21,22]. The questionnaire was divided into four sections reflecting the variety of information required. These include general demographic, anthropometric and lifestyle information, socio-economic information, reproductive information, and physical activity information. The instrument was assessed for relevance and clarity by three experts who provided useful feedback and subsequent approval. The questionnaire was further pretested on 17 participants at General Hospital Lagos Island and appropriate modifications made based on the response and feedback from the participants and interviewers.

There were a mix of interviewers comprising people who could speak English, the local language of the study area, as well as Pidgin English (the Nigerian version of English) which most urban dwellers in Nigeria understand. During the early phase of the study, participants were offered a modest payment or soap; they were informed about this after completing the interview. Some participants indicated that there was no need for these tokens and so this process ceased. In total 26% of participants received a payment or soap. To enhance the quality of data generated, we provided a 2 to 4 hour training session for the interviewers (involving recorded mock interviews) ahead of the study. Interviewers recorded the initial study interviews using digital voice recorders (ICD-PX 333 series). However, some patients expressed concern over privacy of the recordings so not every interview was recorded. We checked all completed questionnaires before the end of each day of data collection and efforts were made to re-contact patients where possible for any detected error or missing information.

**Measurement of socio-economic status:** two variables for educational attainment were defined-educational achievement of the woman herself, and that of her husband (excluding never married women). Respondents with (or those whose husbands had) a Higher National Diploma Certificate (HND) were classified as first-degree holders, while post-secondary education was defined as any training including technical and vocational education leading to an award of certificate following completion of secondary education but falling below HND or first degree. Educational attainment was classified, for analysis, as non-formal/primary, secondary, postsecondary, first degree/HND and >first degree.

Information was collected on the specific job held by the participants and their husbands. Occupational status was defined based on the International Standard Classification of Occupational Status, issue 08 volume 1 (ISCO: 08, vol. 1) [23]. Specific job was defined as the most recent job held for 2 or more years (before diagnosis of breast cancer for cases). Occupational status was assessed based on the participant´s occupation and that of her husband. Because of the small sample size in some occupational groups, respondent´s occupation was classified, for analysis, as unemployed/housewife; elementary/craft/trades occupation; services/sales /clerical workers; professionals/associate professionals/managers. Husband´s occupation was classified as plant/machine operators; craft/related trades; services/sales/clerical support workers; professionals/associate professionals/managers. Income was defined as the total amount of money (in Nigerian naira - ₦) accruing to the respondent or her husband in a month irrespective of the source. Respondent´s and husband´s personal income were both categorised into four groups: < ₦18,000; ₦18,000 - ₦49,000; ₦50,000 - ₦100,000; > ₦100, 000. The lower cut off value (₦18,000) was based on the national minimum wage in 2017 [24].

Data on wealth index was based on property ownership. We collected data on the type of accommodation/tenement the woman lived in (detached rented house and detached personal house, 2-4-bedroom flat, mini flat/apartment, family flat/house, single/double room) and ownership of a private car (by both the respondent and her spouse with the response categories of both owning personal cars, respondent only, shared car, husband only, no car). These two variables were scored from 4 to 0 (in the order of presentation above), summed and categorised into three equal groups as high, middle, and low. Given the potential correlations among the socio-economic variables of interest, we computed a socio-economic index (SEI) - a single indicator that captured the average effect of all the variables. This was derived by converting the dummy variables assigned to each ordinal category of personal educational attainment, husband´s educational attainment, personal occupational status, husband´s occupational status, personal income, husbands/helpers´ income, and wealth index into scores and computing the average sum for each participant. The average sum was split into 4 equal groups categorised as very low, low, high and very high socio-economic index (SEI).

**Statistical analyses:** the differences in distribution of demographic factors and explanatory variables between cases and controls were assessed using t-tests or Mann Whitney U (for non-normally distributed variables) for continuous variables. Categorical data were compared using Chi square (χ^2^) tests. Unconditional binary logistic regression was used to model the relationship between breast cancer and socio-economic status using Statistical Package for Social Sciences (SPSS) version 23. Unadjusted odds ratios and 95% confidence intervals were computed for each SES variable. Multicollinearity for continuous variables was assessed and assumed not to be present if the tolerance value was >0.1 and the variance inflation factor <10 [25]. Pairwise deletion was applied to all missing values since most missing values were <10%. Adjusted models were developed for each SES variable since they emphasize different aspects of SES despite being correlated [24]. For example, while education and income capture knowledge-related and material-based assets, occupation reflects prestige and social standing [24]. However, for intervention purposes (given resource limitations in Nigeria), we further adjusted each SES variable for other SES variables in order to isolate their independent effects.

The relevant explanatory variables were selected based on the existing literature [26]. They were included in the models in 4 stages. The first stage (model 1-minimally adjusted model) comprised the base variables. The base variable (age [as continuous variable), study sites, and ethnicity [Yoruba, Igbo, Niger Deltans, other northern tribes]) were entered first because cases and controls were expected to be similar in age and to represent the urban population of Nigeria. The reproductive variables [reproductive variables comprising parity (continuous variable), age at first pregnancy/birth (AAFB) (continuous variable), menopausal status (pre-menopausal and post-menopausal), total months of breastfeeding-TBF (continuous), age at menarche (AAM) (≤13years and >13years), oral contraceptive use (OCU) (yes and no), and history of induced abortion ((HIA) (yes and no)] were entered next in the second stage (model 2- core 1 model) because of their strong influence on SES [26]. In the 3^rd^ stage (model 3-core-2 model), we additionally adjusted for the effects of body mass index (BMI) (continuous variables), urbanicity (less urbanised, more urbanised), family history of breast cancer (FHBC) (yes and no), alcohol consumption (yes and no), total physical activity (PA) (tertiles). In the 4^th^ stage (model 4) mutual adjustments for other composite SES variables were done. This order of variable adjustments was maintained for SEI (except for the 4^th^ stage which was not applicable) for consistency purpose. Model goodness of fit was checked using the Hosmer and Lemeshow test, as well as assessment of residuals (standardized residual and Cook´s distance statistics).

We explored the modifying effects of menopausal status and age (<50 and ≥50yrs) on SES (based on SEI) using a stratified analysis. All reported p values were based on likelihood ratio tests. Throughout p <0.05 (two-sided) was considered statistically significant. Sensitivity analyses restricted to (1) participants resident within the geographic boundaries of Lagos and Abuja, (2) cases diagnosed within 12 months, (3) controls (patients/visitors) seen in the ophthalmology department were carried out to determine if the excluded participants affected the models substantially.

**Ethical consideration:** the study protocol and data collection instruments were approved by the Ethics Committees of the five participating hospitals (NHA/EC/085/2016; FCT/UATH/HREC/PR/537; ADM/DCST/HREC/APP/1108; NRECC04/04/2008; SUB/GHL/1288/19) described earlier as well as those of Newcastle University, United Kingdom (1031/2016) and Lagos State Health Services Commission, Lagos, Nigeria (LSHSC/2222/Vol.XIX/48). Informed consent was obtained in writing from all participants using a consent form specifically prepared for the purpose. In the case of the aged and illiterate participants, it was obtained by proxy through a relative they trusted and designated to act on their behalf. The questionnaires did not carry the name of the patients nor any other information capable of identifying them. Unique identification codes were generated for each participant.

## Results

**Figure 1:**
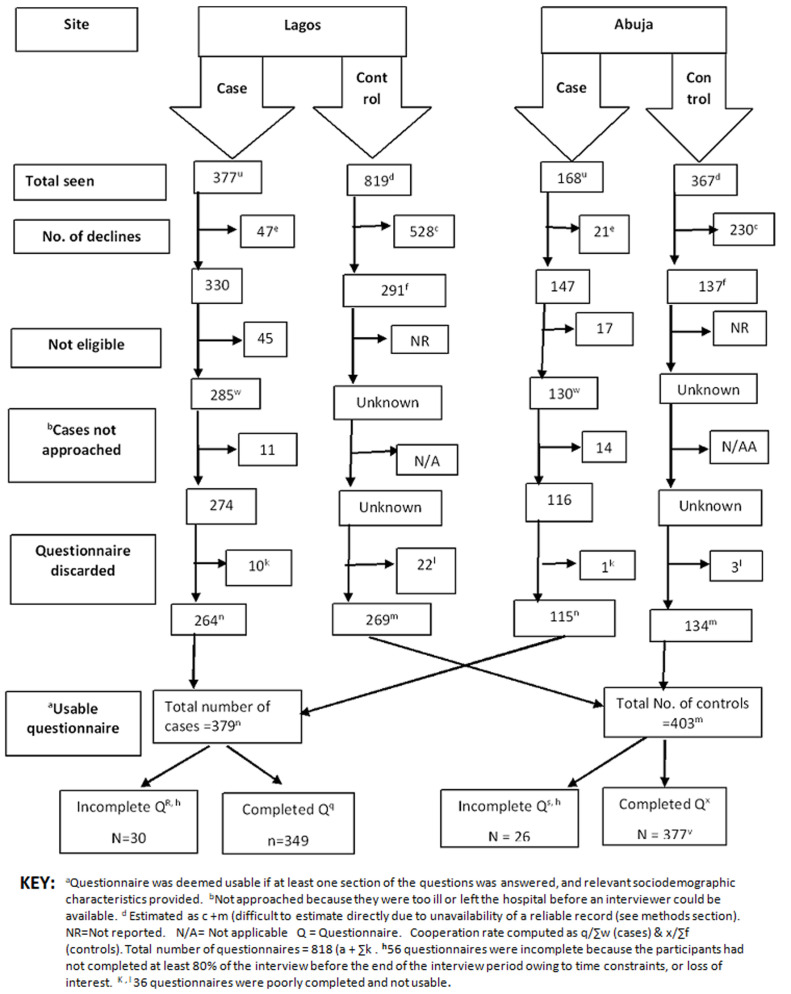
recruitment flow chart

A total of 372 cases and 403 controls were recruited (Figure 1). The cooperation rate (the number of completed interviews among eligible participants) [27] was 84.1% for cases and 88.1% estimated for controls assuming all potential controls who declined were not eligible (Figure 1). Descriptive analyses (Table 1) show that cases did not differ significantly from controls with respect to age, ethnicity, marital status, age at first birth, age at menarche, oral contraceptive use, body mass index, total months of breastfeeding, parity, or history of induced abortion. Significant differences in proportions between cases and controls were observed with respect to urbanicity, family history of breast cancer, menopausal status, income, education, and occupational status. The proportion of participants with higher levels of educational attainment, occupational status and income was higher for controls than cases (Table 2).

**Table 1:** Participants' characteristics

Characteristics	Control		Case		^∞^P
	n (%)	Missing*	n (%)	Missing*	
**Age**					0.583
< 50.00 yrs.	247(61.3)		225 (59.4)		
≥ 50.00 yrs.	156 (38.7)		154 (40.6)		
Mean ± SD	46.8 ± 10.8		47.1 ± 10.7		0.556^β^
**Ethnicity**		2(0.5)		1(0.3)	0.098
Yoruba	192 (47.9)		155 (41)		
Igbo	100 (24.9)		128 (33.9)		
Hausa / Fulani	14 (3.5)		13 (3.4)		
Niger Deltans	51 (12.7)		42 (11.1)		
Other Northern Tribes	44 (11)		40 (10.6)		
**Marital status**		4(1)		2(0.5)	0.545
Never Married	33 (8.3)		36 (9.5)		
Widowed	32 (8.0)		26 (6.9)		
Divorced / separated	9 (2.3)		14 (3.7)		
Married	325 (81.5)		301 (79.8)		
**Religion**		4(1)		2(0.5)	0.145
Christianity	315 (78.8)		310 (82.9)		
Islam	85 (21.3)		64 (17.1)		
**Ever consumed alcohol?**		4(1)		0(0)	0.894
No	235 (58.9)		225 (59.4)		
Yes	164 (41.1)		154 (40.6)		
**Family history of BC (FHBC)**		3(0.7)		0(0)	0.002
No	381 (95.3)		339 (89.4)		
Yes	19 (4.8)		40 (10.6)		
**Urbanicity of area of residence**	1(0.2)		1(0.3)	0.007	
More urbanized	348 (86.6)		299 (79.1)		
Less urbanized/rural	54 (13.4)		79 (20.9)		
**Body mass index-BMI (Kg/M^2^)**		36(8.9)		37(9.8)	0.265^ᵟ^
Median (IQR)	27.77 (7.29)		26.76 (7.26)		
**Parity**		8(2)		1(0.3)	
Median (IQR)	3.0 (2)		3.0(2)		0.09
**Total months of breast Feeding (TBF)**	11(2.7)		6(1.6)		
Median (IQR)	36(36)		36.5(41)		0.61
					
**Age at menarche (AAM)**		19(4.7)		11(2.9)	0.57
≤ 13yrs	127 (33.1)		129 (35.1)		
>13yrs	257 (66.9)		239 (64.9)		
**Menopausal Status**		Remove missing value		Remove missing value	0.02
Premenopausal	229 (56.8)		161 (42.5)		
Unknown/artificial*	20 (5.0)		64 (16.9)		
Post- menopausal (Natural	154 (38.2)		154 (40.6)		
**Ever used oral contraceptives (OCU)?**	14(3.5)		12(3.2)	0.26	
No	312 (80.1)		282 (76.8)		
Yes	77 (19.8)		85 (23.2)		
**Age at first birth (AAFB)**		58(14.4)		49(12.9)	
Mean ± SD	25.5 ± 4.8		25.3± 5.1		0.577^β^
**Physical activity-PA (MET-hr/wk)**		23 (5.7)		16 (4.2)	0.082
< 128.20	134 (36.9)		112 (29.5)		
128.20 - 184.29	118 (32.5)		131(34.5)		
≥184.30	111 (30.6)		137 (36.1)		

ᵟ M-W=Mann-Whitney U test (p value); SD = standard deviation; ^∞^differences between cases and controls based on LRT (likelihood ratio test). *Excluded (cases with contradictory answers /participants whose menstrual flow ceased as a result of other reasons apart from the natural process). ^β^Based on t-test of independent samples. *Missing values includes 'not applicable'

**Table 2:** relationship between socio-economic measures, and breast cancer risk (unadjusted result)

	Control-n (%)	M*	Case-n (%)	M*	OR (95% CI)	P^η^
**Education**		3 (0.7)		0 (0)		<0.001
Non-formal/primary	37 (9.3)		63 (16.6)		1.00 (ref)	
Junior/senior secondary	96 (24)		109 (28.8)		0.67 (0.41, 1.09)	
Post-secondary	73 (18.3)		71 (18.7)		0.57 (0.34, 0.96)	
1^st^ degree/HND	134 (33.5)		110 (29)		0.48 (0.30, 0.77)	
>1^st^ degree	60 (15)		26 (6.9)		0.25 (0.14, 0.47)	
**Husband's education**		45 (11.2)		42 (11.1)		0.197
Non-formal/primary	32 (8.9)		39 (11.6)		1.00 (ref)	
Secondary	88 (24.4)		106 (31.6)		1.14 (0.64, 2.03)	
Post-secondary	34 (9.4)		30 (9.0)		0.85 (0.42, 1.72)	
1^st^ degree/HND	137 (38.1)		117 (34.1)		0.87 (0.49, 1.54)	
>1^st^ degree	69 (19.2)		43 (12.8)		0.62 (0.33, 1.18)	
**Respondents' income**		28 (6.9)		30 (7.9)		<0.001
< ₦18,000	71 (18.9)		100 (28.7)		1.00 (ref)	
₦18,000 - ₦49, 000	106 (28.3)		128 (36.7)		0.86 (0.58, 1.28)	
₦50,000 - ₦100,000	123 (32.8)		77 (22.1)		0.44 (0.29, 0.67)	
> ₦100,000	75 (20.0)		44 (12.6)		0.42 (0.26, 0.67)	
**Husband's income^eβ^**		139 (34.5)		112 (29.6)		0.026
< ₦50,000	72 (27.7)		98 (27.3)		1.00 (ref)	
₦50,000 - ₦100,000	92 (34.8)		92 (34.8)		0.74 (0.49, 1.13)	
> ₦100,000	100 (37.9)		76 (28.5)		0.56 (0.37, 0 .86)	
**Wealth index**		15 (3.7)		11 (2.9)		<0.001
Very low/low	98 (25.3)		138 (37.5)		1.00 (ref)	
Middle	152 (39.2)		146 (39.7)		0.68 (0.48, 0.96)	
High/very high	138 (35.5)		84 (22.8)		0.43 (0.30, 0.63)	
**Occupation**		2 (0.5)		2 (0.5)		0.001
Unemployed/housewife	22 (5.5)		38 (10.1)		1.00 (ref)	
Elementary/craft/trades	45 (11.2)		42 (11.1)		0.54 (0.28, 1.06)	
Services /sales/clerks	171 (42.6)		176 (46.7)		0.60 (0.34, 1.05)	
Professionals	163 (40.6)		121 (32.1)		0.43 (0.24, 0.76)	
**Husbands' occupation^β^**		64 (15.9)		56 (14.8)		0.087
Plant, machine operators	45 (13.3)		63 (19.5)		1.00 (ref)	
Services/sales/clerks	110 (32.4)		102 (31.6)		0.66 (0.42, 1.06)	
Professionals^g^	184 (54.3)		158 (48.9)		0.61 (0.40, 0.95)	
**Socio-economic index**		2 (0.5)		0 (0)		0.001
Very low	77 (16.2)		109 (28.8)		1.00 (ref)	
Low	88 (21.9)		111 (29.3)		0.89 (0.60, 1.34)	
High	111 (27.7)		75 (19.8)		0.48 (0.32, 0.72)	
Very high	125 (31.2)		84 (22.2)		0.48 (0.32, 0.71)	

* Missing values; ^g^includes associate professionals and managers; ^β^Missing values include 'not applicables'; ^η^p for trend; ^e^Husbands include any other source of income (for unmarried women)

The multivariable analysis shows a decreasing trend in breast cancer risk as personal educational attainment increased (Table 3). The estimates attenuated but remained significant following adjustment for the effect of the core variables (p for trend = 0.003). However, the statistical significance disappeared after adjustments for other SES variables (p for trend = 0.123) (Table 3). A significant association with breast cancer was not observed for husbands´ educational attainment in any of the models (Table 3).

**Table 3:** relationship between SES measures and breast cancer risk (multiple regression)

SES variable categories	Model 1	Model 2	Model 3	Model 4
	^a^OR (95% CI)	^b^OR (95% CI)	^c^OR (95% CI)	^d^OR (95% CI)
**Educational attainment**				
Non-formal/primary	1.00 (ref)	1.00 (ref)	1.00 (ref)	1.00 (ref)
Junior/senior secondary	0.61 (0.37, 1.03)	0.58 (0.32, 1.04)	0.77 (0.41, 1.48)	0.77 (0.38, 1.55)
Post-secondary	0.52 (0.30, 0.90)	0.55 (0.29, 1.04)	0.79 (0.39, 1.58)	1.17 (0.51, 2.71)
1^st^ degree/HND	0.41 (0.24, 0.69)	0.35 (0.18, 0.69)	0.47 (0.23, 0.99)	0.76 (0.30, 1.99)
>1^st^ degree	0.22 (0.12, 0.42)	0.20 (0.09, 0.44)	0.21 (0.09, 0.53)	0.37 (0.12, 1.16)
P for trend	<0.001	0.001	0.003	0.123
**Husband's education**				
Non-formal primary	1.00 (ref)	1.00 (ref)	1.00 (ref)	1.00 (ref)
Secondary	1.14 (0.64, 2.02)	0.91 (0.47, 1.75)	1.31 (0.63, 2.71)	1.38 (0.62, 3.05)
Post-secondary	0.85 (0.42, 1.72)	0.58 (0.25, 1.33)	0.86 (0.35, 2.15)	1.08 (0.39, 2.99)
1^st^ degree/HND	0.87 (0.49, 1.54)	0.76 (0.40, 1.51)	1.18 (0.56, 2.49)	1.97 (0.82, 4.71)
>1^st^ degree	0.62 (0.33, 1.18)	0.51 (0.24, 1.08)	0.71 (0.31, 1.64)	1.25 (0.47, 3.35)
P for trend	0.197	0.288	0.307	0.299
**Respondents' income**				
< ₦18,000	1.00 (ref)	1.00 (ref)	1.00 (ref)	1.00 (ref)
₦18,000 - ₦49, 000	0.83 (0.55, 1.25)	0.99 (0.61, 1.61)	1.05 (0.63, 1.76)	1.06 (0.62,1.83)
₦50,000- ₦100,000	0.44 (0.28, 0.67)	0.40 (0.24, 0.67)	0.40 (0.23, 0.70)	0.45 (0.24, 0.85)
> ₦100,000	0.39 (0.24, 0.65)	0.35 (0.19, 0.66)	0.37 (0.19, 0.72)	0.44 (0. 20,1.00)
P for trend	<0.001	< 0.001	< 0.001	0.014
**Husband's income^e^**				
< ₦50,000	1.00 (ref)	1.00 (ref)	1.00 (ref)	1.00 (ref)
₦50,000 - ₦100,000	0.78 (0.51, 1.20)	0.66 (0.40, 1.10)	0.68 (0.38, 1.20)	0.68 (0.37, 1.25)
> ₦100,000	0.57 (0.36, 0.89)	0.44(0.25, 0.77)	0.44 (0.24, 0.82)	0.58 (0.28, 1.20)
P for trend	0.044	0.015	0.032	0.307
**Wealth index**				
Very low/low	1.00 (ref)	1.00 (ref)	1.00 (ref)	1.00 (ref)
Middle	0.68 (0.48, 0.97)	0.58 (0.40, 0.90)	0.58 (0.37, 0.92)	0.56 (0.33, 0.94)
High/very high	0.39 (0.26, 0.57)	0.40 (0.23, 0.62)	0. 42 (0.25, 0.72)	0.70 (0.35, 1.39)
P for trend	< 0.001	<0.001	0.005	0.082
**Occupation**				
Unemployed^β^	1.00 (ref)	1.00 (ref)	1.00 (ref)	1.00 (ref)
Elementary/craft/trade	0.58 (0.29, 1.14)	0.68 (0.30, 1.54)	0.52 (0.23, 1.19)	0 .56 (0.20, 1.50)
Services/sales/clerks	0.64 (0.36, 1.14)	0.66 (0.32, 1.35)	0.67 (0.27, 1.69)	0.69 (0.28, 1.70)
Professionals^g^	0.45 (0.25, 0.80)	0.50 (0.24, 1.04)	0.69 (0.31, 1.57)	0.76 (0.29, 1.98)
**Husbands' occupation**				
Machine operators	1.00 (ref)	1.00 (ref)	1.00 (ref)	1.00 (ref)
Services/sales/clerks	0.59 (0.36, 0.96)	0.58 (0.32, 1.07)	0.59 (0.30, 1.13)	0.67 (0.36, 1.23)
Professionals^g^	0.62 (0.39, 0.97)	0.65 (0.37, 1.13)	0.80 (0.42, 1.52)	0.55 (0.26, 1.15)
**Socio-economic index**				
Very low	1.00 (ref)	1.00 (ref)	1.00 (ref)	NA
Low	0.84 (0.55, 1.27)	0.70 (0.42, 1.16)	0.74 (0.42, 1.29)	
High	0.48 (0.32, 0.74)	0.45 (0.27, 0.74)	0.51 (0.29, 0.89)	
Very high	0.46 (0.30, 0.70)	0.41 (0.24, 0.69)	0.46 (0.26, 0.80)	
P for trend	<0.001	0.002	0.028	

a Adjusted for age, study sites, ethnicity; ^b^Additionally adjusted for AAFB, parity, menopausal status, AAM, OCU; ^c^Additionally adjusted for-BMI, urbancity, alcohol use, FHBC, total PA. ^d^Mutually adjusted for other socio-economic variables; ^e^Husbands include any other source of income (for umarried women). ^g^Includes associate professionals. ^β^Includes housewives

There was also evidence of decreasing risk of breast cancer as personal income and husband´s income, increased (Table 3). The linear relationship remains relatively stable for personal income (p for trend <0.001) but attenuated for husband´s income (p for trend = 0.032) following adjustments for the effects of the base and core variables (Table 3, Model 3). However, while the increasing risk of breast cancer with increasing personal income remained significant (p for trend = 0.014) after adjustments for the effects of occupational, educational and wealth status, the significant association between breast cancer and husband´s income disappeared (p = 0.307) after adjustments for other SES variables. Similarly, the increasing risk of breast cancer associated with increasing wealth index following adjustments for the effects of the base variables, remained significant after adjustments for the effects of the core variables (p for trend= 0.005). This observed trend however disappeared after adjustments for occupational, educational and income status (p = 0.082). No measure of occupational status was significantly associated with a reduced risk of breast cancer after adjustments for the core variables. Moreover, the increasing trend of breast cancer associated with increasing SEI, remained significant (although attenuated) after adjustments for the effects of the base and core variables (p for trend = 0.028) (Table 3). The risk of breast cancer associated with high SES was more marked among younger women than older women (Table 4). The results of the 4 sensitivity analyses (Annex 1) conducted showed consistency with the results shown in Table 2.

**Table 4:** relationship between socio-economic status and the risk of breast cancer stratified by menopausal status and age

Socio-economic index	Menopausal status stratification	
	Pre-menopausal	Post-menopausal
	OR (95% CI)	OR (95% CI)
Low	1.00 (ref)	1.00 (ref)
Middle	0.61 (0.30, 1.24)	0.83 (0.41, 1.67)
High	0.40 (0.19, 0.87)	0.49 (0.24, 0.99)
P for trend	0.066	0.117
	**Age stratification**	
	**Age < 50yrs**	**Age ≥ 50yrs**
Low	1.00 (ref)	1.00 (ref)
Middle	0.64 (0.32, 1.29)	0.92 (0.44, 1.91)
High	0.46 (0.22, 0.95)	0.48 (0.22, 1.02)
P for trend	0.105	0.108

Adjusted for study site and ethnicity, AAFB, parity, menopausal status, AAM, TBF, oral contraceptive use, HIA, BMI, alcohol use, FHBC, PA and mutual adjustments for menopausal status and age as applicable

## Discussion

This study found that a reduced risk of breast cancer was associated with higher personal educational attainment, income, wealth and socio-economic index after adjusting for other explanatory variables. The association was only partially accounted for by other traditional risk factors especially age, menopausal status, age at first birth, BMI, physical activity, and family history of breast cancer (Annex 2). Occupational status was not significantly associated with a reduced risk of breast cancer. Measurements based on husband´s SES were not independently associated with risk of breast cancer.

Our findings were somewhat surprising given the patterns of association between breast cancer and SES that have been observed in HIC where high educational attainment [4,28] and occupational status [4,29] were associated with an increased risk of breast cancer. Notably, more than 70% of breast cancer cases in Nigeria are diagnosed at advanced stage compared to <30% in England and Norway [30-32]. Moreover, there are studies in the USA [5], Denmark [21], Puerto Rico [33], Iran [34], and Brazil [35,36] that have reported a reduced risk of breast cancer with increased levels of income and education as observed in our study. The result of the Danish study, however, was more marked among postmenopausal than premenopausal women contrary to our finding. This could be attributed to demographic differences associated with population age distribution. Moreover, the distribution of educational attainment in our study was consistent with that observed in a previous population based Nigerian study, although the role of education in breast cancer was not part of the objectives of that study [19]. Our findings, however, were not consistent with two other previous African studies which explored the role of SES in breast cancer based on educational attainment [37,38]. These studies were limited by the number of explanatory variables adjusted for. The sample size in one of the studies [39], however, was higher than our sample but similar to that of a previous Nigerian study with educational distribution consistent with ours [19].

The relationship between income and the risk of breast cancer has not been previously explored in any previous indigenous study to the best of our knowledge. However, our finding with respect to the role of wealth index was consistent with the reduced risk of breast cancer associated with increased property index in a Tanzanian study [14]. Wealth or property index could serve as a proxy measure of income.

The consistency of our findings with the case control study in the USA [5] and the cohort study in Denmark [21] is notable since the breast cancer types in those studies share similar characteristics (advanced stage at diagnosis) with that prevalent in Nigeria and other black African populations [39,40]. Previous indigenous studies have shown that high prevalence of advanced cases at diagnosis is associated with low socio-economic [41,42]. There is also evidence that other characteristics of breast cancer predominantly seen among cases in Nigeria (such as younger age at presentation, high proportion of oestrogen negative and triple negative phenotypes) tend to be associated with low SES [43-45] compared to most cases in white populations which are predominantly postmenopausal, oestrogen receptor positive and predominantly present at early stage (often as a result of participation in mammographic screening commonly observed among women of higher SES) [45-47]. We could not stratify our analysis by oestrogen receptor status because in addition to the small sample size, the availability of the data (Annex 3) might be influenced by the SES measures being investigated.

In addition to the potential role of stage at diagnosis and oestrogen receptor status distribution, our findings could be potentially explained by the fact that women of high socio-economic status tend to have a higher-level of awareness which increases their capacity to reduce exposures to other breast cancer-predisposing factors such as physical inactivity, alcohol consumption (which we adjusted for) and postmenopausal obesity (which we did not have separate information on) [5,48-50]. Their good health-seeking behaviour increases the chances of timely removal of precancerous breast lumps while reducing the incidence of advance stage breast cancers [50]. On the other hand, low income earners are more likely to present late for timely diagnoses (as they tend to explore cheaper options first) as well as reside within city locations prone to breast cancer-associated environmental pollutants [51].

The stronger role of income compared to other SES measures in predicting breast cancer emphasises its stronger role in health protection in low socio-economic settings compared to observations in high income countries where education has been identified as playing a greater role [52,53]. Income has been described as the best single indicator of material living standards, as well as the most direct measure of material resources, thought to have a “dose-response” association with health [52]. The effect of occupation in our study should not be surprising because its effect tends to be weaker and sometimes inconsistent with those of education and income, possibly due to misclassification bias, or effect of unadjusted work-based exposures [4,21,22]. Unfortunately, the independent role of occupation has not been reported in any previous indigenous study to the best of our knowledge. Hence, comparison of findings was not possible.

Our study is expected not only to generate discussion (e.g. to rethink and broaden preventive policies tailored towards the peculiarities in sub-Saharan African countries), but also to suggest the need to consider the socio-economic empowerment of women through improved educational and income/ wealth creation opportunities (mostly targeted at women of low SES) as part of breast cancer preventive strategy in Nigeria. As our findings suggest, socio-economic status of women in this context cannot be substituted with those of their husbands. Alternatively, in the short term, the cost of screening, diagnoses and treatment of breast cancer could be subsidised alongside awareness intervention targeted at women of low SES. This will encourage early presentation, improve health seeking behaviour and reduce the incidence of advanced stage invasive breast cancers associated with low SES. These recommendations, however, should be implemented bearing in mind the potential breast cancer risk associated with high SES as has been reported in HIC. All the same, given the discrepancy with the findings of other previous studies, we recommend a confirmation of the findings based on more methodologically robust hypothesis-driven studies where all relevant SES indicators, confounders and mediators are considered at the design stage.

Our study is the first to explore the relationship between SES and risk of breast cancer in Africa based on a range of individual-level SES measures including education, income, occupation, and wealth index which were selected a priori. We adjusted for several explanatory variables (breast feeding, parity, age at first birth, physical activity) which were not accounted for in previous studies. Nevertheless, our study has limitations. Breast cancer patients of high SES might seek treatment overseas (and perhaps in private hospitals). That could create the potential for overrepresentation of women of high SES among our controls. Hence, giving the impression that high SES is protective. While we have no data on breast cancer patients who might seek treatment overseas, the preference for private hospital visits has been reported among ophthalmology patients (controls) of high SES as well [54]. Moreover, we explored the representativeness of our controls to the urban female population of Nigeria by comparing the wealth profile, age at first birth, the mean number of children among our controls to those of Nigerian women residing in urban areas, Lagos and Abuja to the National demographic health survey 2013 data (Annex 4, Annex 4 (suite) and Annex 5). Interestingly, we observed similarities in their distributions. We also observed a similarity between the distribution of the educational profile of our controls and that reported in a previous population-based case-control study in Nigeria [19]. While this may make a case for generalisability, we do not know the extent to which they reflect the experience of less urbanised communities and regions of Nigeria especially in the core North with different demographic profiles. Moreover, our inability to obtain specific data on the profile of urban women who seek treatment in places other than public hospitals, should be considered in interpreting our findings. Despite our efforts to recruit participants in the order in which they arrived or were seated in the clinics, the potential for selection or participation bias may not be ruled out.

## Conclusion

This study shows a strong association between higher SES and lower breast cancer risk among Nigerian women. The findings contrast predominant observations in high income countries and indicate the need for preventive policies based on local experience. While we recommend that the findings be confirmed in future studies, it suggests the need to consider socio-economic improvement of women as part of breast cancer prevention in Nigeria alongside intervention to educate lower SES women about breast cancer risk factors and need to present early to health services (and providing access to facilities for these women).

### What is known about this topic

- *Most studies especially in high income countries (HIC) have shown that high socio-economic status is associated with an increased risk of breast cancer;*
- *Among populations with predominantly high risk or advance metastatic breast cancer, a few studies in high income countries have shown that low socio-economic status was associated with an increased risk of breast cancer;*
- *The findings on the relationship between socio-economic status and breast cancer risk in sub-Saharan Africa based on the available studies have been inconsistent*.

### What this study adds

- *Our study is the first African study to explore the relationship between socio-economic status and the risk of breast cancer based on multiple socio-economic measures-occupational status, educational attainment, income, wealth index and socio-economic index;*
- *The socio-economic status of women cannot effectively be substituted with those of their husbands/partners with respect to any socio-economic intervention towards the prevention of breast cancer in Nigeria;*
- *Our study (subject to confirmation) might query the assumption that high socio-economic status increases the risk of breast cancer in Nigeria (and other sub-Saharan African countries) similar to the experience in high income countries. Hence, the study will provide a basis for more investigations on the topic*.

## Annexes

**Annex 1**: sensitivity analyses based on the fully adjusted models for educational attainment and income measures (PDF-425 kb)

**Annex 2**: relationship between socio-economic status (based on socio-economic index) and breast cancer-explanatory variable selection criteria (PDF-401 kb)

**Annex 3**: available information on immunohistochemistry record of breast cancer patients (PDF-528 kb)

**Annex 4**: external data (NDHS 2013) on wealth distribution among women 15-49 years (PDF-406 kb)

**Annex 4 (suite)**: external data (NDHS 2013) on wealth distribution among women 15-49 years (PDF-417 kb)

**Annex 5**: updated data (NDHS 2018) on wealth distribution among women 15-49 years (PDF-407 kb)
